# Macroscopic and histological analyses of cremated remains from the Imperial Roman necropolis of La Cona (1^st^ cent. BCE-1^st^ cent. CE, Teramo, Italy)

**DOI:** 10.1371/journal.pone.0345498

**Published:** 2026-04-22

**Authors:** Alessia Galbusera, Stefano Magri, Owen Alexander Higgins, Martina Trevisan, Vito Giuseppe Prillo, Massimo Vidale, Vincenzo Torrieri, Stefano Benazzi, Luca Bondioli, Alessia Nava, Melania Gigante

**Affiliations:** 1 Department of Odontostomatological and Maxillofacial Sciences, Sapienza University of Rome, Rome, Italy; 2 Department of Cultural Heritage, University of Bologna, Ravenna, Italy; 3 Department of Cultural Heritage, University of Padua, Padua, Italy; 4 Soprintendenza Archeologia, Belle Arti, Paesaggio, L’Aquila-Teramo, Italy; 5 Institute of Geological Sciences, Polish Academy of Sciences, Krakόw, Poland; Institute for Anthropological Research, CROATIA

## Abstract

Cremation was one of the most widespread funerary practices between the Bronze Age and Late Antiquity in Mediterranean societies. Despite its archaeological relevance, the analysis of cremated human remains has long been limited by extensive thermal alteration, which hampers the reconstruction of individuals’ biological profiles. However, in recent years, renewed methodological attention has highlighted the potential of cremated remains to yield reliable bioarchaeological information. In this study, we apply an integrated macro- and microscopic approach to the analysis of 26 cremations from the Imperial Roman necropolis of La Cona (central Italy). Macroscopic examination was combined with bone histology to discriminate human from non-human bone fragments, assess heat-induced taphonomic alterations at the microstructural level, and estimate age at death through histomorphometric parameters. Although the sample size is necessarily limited by preservation and selection constraints, the results demonstrate the effectiveness of histological analysis for improving taxonomic attribution and refining age-at-death estimation in cremated assemblages. More broadly, this study highlights the methodological value of integrating macroscopic and microscopic evidence for the bioarchaeological investigation of Roman-period cremations.

## Introduction

The ritual of cremation, consisting of burning the body of the deceased was a widespread funerary practice among ancient human populations. This practice has been widely employed throughout history and is still practiced today by many cultures worldwide [1–3].

The earliest evidence of cremation as a funerary practice in Europe dates back to the Mesolithic period [4–6]. Still, it was not until the Bronze Age that it became the predominant ritual, reaching its peak with the so-called *Urn Fields culture* [7,8].

Peninsular Italy mirrors the broader European trend, exhibiting a gradual transition from inhumation to cremation that commenced in the Middle Bronze Age [7,9,10]. In the Early Iron Age, several regional cultural horizons were notable for their cremation rites. One such example is the Latial culture (c. 10^th^-8^th^ centuries BCE) in Latium (central Italy) [11–13]. The Latial people employed distinctive hut-shaped urns for the storage of cremated remains, frequently interring them within cylindrical pits. The urned cremated remains were often accompanied by miniaturized grave goods, including spearheads, knives, and razors, which may reflect symbolic or resource-related choices [11,14].

Concurrently, the Villanovan culture (c. 9^th^–8^th^ centuries BCE) also featured cremation as a central funerary ritual. The cremated remains were placed in biconical urns, sometimes accompanied by bowls or helmets, and deposited within pit graves. These burials were often accompanied by grave goods that indicate the deceased’s social status and gender roles [15,16]. Comparable patterns of cremation have been observed among other Italic groups in central and northern Italy [7,17,18] although inhumation persisted in some areas, suggesting regional diversity rather than cultural uniformity [7].

From the 8^th^ century BCE onwards, the arrival of Greek settlers in Magna Graecia and Sicily brought with it new funerary practices. These rites, often imbued with symbolic references to the world of the banquet, centered primarily on cremation and stood in marked contrast to the prevailing inhumation traditions of the indigenous Italic communities [19–22].

In Rome, cremation was already part of the funerary repertoire, though it coexisted with inhumation [23]. It was only in the early Imperial period, from the second half of the 1^st^ century BCE, that cremation emerged as the dominant funerary custom across the Italian peninsula and within the wider provinces of the Roman Empire [24]. For the Romans, cremation was not merely a practical method of corpse disposal [25]; rather, it carried profound ritual significance, likely within the belief that the soul departed the body with the final breath [23]. The inclusion of items such as meal remains or animal astragali on the pyre further supports this ritual dimension [23,25,26].

The analysis of ancient cremated remains provides valuable insights to reconstruct circumstances and behaviors surrounding death and the funerary sphere [27]. The secondary deposition of selected cremated remains – so-called *ossilegio*– in the tomb [28–30] along with the placement of grave goods and animal remains [31] provides evidence of specific ritual behaviors. Furthermore, research on the visible effects of combustion, such as chromatic alteration [32], fractures [33], and mechanical modifications [29,34] has greatly advanced our understanding of heat-induced changes in bone. However, these macroscopic features can only provide a partial and approximate reconstruction [35,36] of some parameters of the cremation process (temperature and oxidation levels and duration of the cremation).

Moreover, the destructive nature of the burning process often limits the study of incinerated human remains, making it difficult—if not sometimes impossible—to reconstruct both the biological profile of the individual and the mortuary practices employed [37]. Nevertheless, a high degree of macroscopic alteration does not necessarily correspond to poor preservation of the bone microstructures [38], although morphological and dimensional modifications are frequently observed [38–41].

In recent years, some of these limitations have been overcome using histological and histomorphometric approaches in the study of cremated remains [19,41–43]. Osteological research has demonstrated that bone microstructures can survive the combustion process, as first observed by Kerley [44] and later confirmed by Kerley & Ubelaker [45]. Bone tissues’ histological analysis has proven to be a reliable tool for distinguishing between human and faunal remains, particularly in commingled assemblages [19,46–52], as bone microstructure exhibits species-specific architectural features. For instance, the presence or absence of plexiform bone, the occurrence of osteon banding (*i.e.*, a linear arrangement of the secondary osteons, as defined by Mulhern & Ubelaker [47] and the variations in size and geometry of the Haversian system features [19,53–57]have been identified as useful indicators of interspecies differences. Moreover, cortical bone histology can provide valuable information on age-related changes in bone microstructures, facilitating age-at-death estimation [19,40,44,45,58]. Despite these considerations, the impact of ageing on bone microstructures in cremated remains, particularly those from archaeological contexts, remains a matter of debate [53,59–69].

The present study investigates skeletal remains from 26 secondary cremations from the Imperial Roman necropolis of La Cona (1^st^ century BCE – 1^st^ century CE, Teramo, Italy), combining macro- and microscopic analysis. Due to the lack of previous bioarcheological studies and the fragmentary nature of the cremated sample, the La Cona necropolis served as an ideal case study for a multi-scalar investigation on ancient cremations.

After macroscopic observations, bone histology and histomorphometry were applied to evaluate heat-induced alterations in bone microstructure, distinguish human from non-human fragments within cremation contexts, estimate age-at-death, and assess osteon shape and size variation, throughout age, across the cremated assemblage. Moreover, it provided new insights into mortuary practices at the necropolis of La Cona.

## Materials and methods

### The Imperial Roman necropolis of La Cona

**Fig 1:**
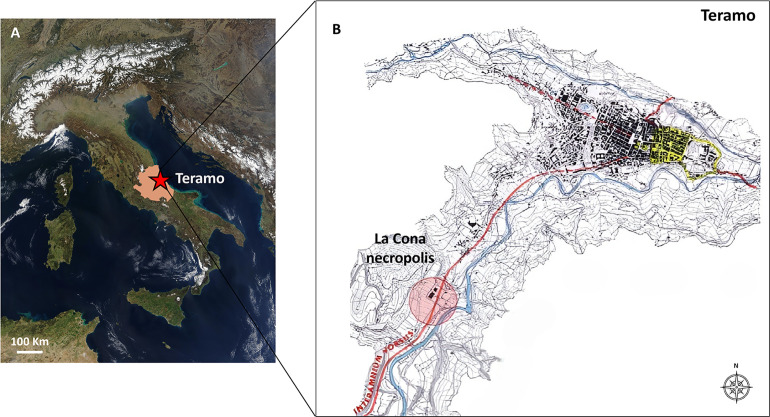
Geographical location of the city of Teramo (Abruzzo, Central Italy). Map showing the geographical location of Teramo, in the Abruzzo region (from NASA Visible Earth project – credits to Jacques Descloitres, MODIS Rapid Response Team, NASA/GSFC).

The necropolis of La Cona is located near the modern town of Teramo (Abruzzo region, Central Italy) (Fig 1). The earliest use of the site as a burial ground dates back to the Early Iron Age [70]. From the 1^st^ century BCE onwards, during the Roman domination of the Picenum territory, cremation burials began to appear in the funerary ritual previously characterized by inhumations only. A distinctive feature of the necropolis is the continuity of its funerary use: Roman period cremations respected the spaces of protohistoric inhumations, avoiding any overlap or disturbance [71].

The site was discovered by chance during urban planning in 1961 and subsequently investigated by the *Soprintendenza Archeologia, Belle Arti e Paesaggio per le province di L’Aquila e Teramo* through two major excavation campaigns: the first, between the 1970s and 1980s, and the second, between 2000 and 2020 [71–73]. Archaeological investigations uncovered a significant number of both primary (*busta sepulcra*) and secondary cremations, where burnt bone remains were collected after the cremation process and interred in ceramic or glass urns or in simple pits dug into the ground, often accompanied by grave goods [70]. The practice of cremation was prevalent at La Cona until the conclusion of the 2^nd^ century CE. During this period, the traditional funerary rite underwent a gradual transition, giving way to a revival of the inhumation *alla cappuccina*, *i.e.,* the burial of the deceased in a pit covered with tiles. The necropolis of La Cona was definitively abandoned in the 3^rd^ century CE and subsequently plundered in medieval times [70].

This study focuses exclusively on secondary cremations from the Imperial Roman period of La Cona necropolis, which is the most documented in the burial area [70]. The analyzed sample consists of 26 funerary contexts, dated between the 1^st^ century BCE and the 1^st^ century CE [70].

The odontoskeletal collection is currently stored at the University of Padua, Department of Cultural Heritage, under the supervision of one of the authors (MV). All permissions were obtained for the described study, which complied with all relevant regulations.

#### Macroscopic analysis of the human sample.

Macroscopic analysis of the cremated remains was conducted following standard protocols [32] (S1 Table). Distinctions between human and faunal remains were based on morphological and morphometric differences in the cortical and inner bone surfaces [52,63,74]. The bone fragments were dry-cleaned using wooden sticks and tweezers to remove any surface residues before any observation was done. The Minimum Number of Individuals (MNI) for the human sample was estimated based on anatomical features, including bone repetitions, robustness, and weight [29,75,76]. Information on recovery and post-excavation procedures, including sieving strategies and mesh size, was not consistently available for all contexts; therefore, their potential impact on fragmentation patterns and weight-based estimates could not be systematically assessed and is acknowledged as a limitation of the study. Color alterations of the skeletal remains were recorded according to Shipman et al. [32] and Walker et al. [77]. This system was selected to ensure comparability with previous archaeological and forensic studies of cremated remains; however, its limitations are well recognized, particularly the overlap between colour categories and the subjectivity involved in linking colour to specific burning temperatures. Accordingly, colour observations in this study were used as general indicators of burning conditions rather than as precise proxies for pyre temperature. The cremated remains attributed to each burial were weighed [29,78,79].

Estimation of age at death was carried out by following the criteria described by Buikstra & Ubelaker [80] for the adults and by AlQahtani et al. [81] for the sub-adults. Biological sex determination was based on osteometric analysis [76] and morphometric observations of cranial and pelvic bones [82].

##### Macroscopic analysis of the faunal sub-sample.

The faunal remains were identified at both taxonomic and anatomical levels using standard reference works for mammals, birds, and mollusks [83]. Quantitative analysis included counting the Number of Identifiable Specimens (NISP) by species and estimating the Minimum Number of Individuals (MNI), according to the method proposed by Bökönyi [83]. Precise age-at-death estimation was conducted only for pig (*Sus domesticus*) remains following Bull & Paine [84]. In contrast, for caprine, a general age estimation was inferred from bone size and morphology. Species differentiation between sheep (*Ovis aries*) and goats (*Ovis* vel *capra*) was attempted through morphological criteria [85] and biometric analysis [86].

Finally, color alterations in faunal remains were analyzed following the methodology outlined by Shipman et al. [32].

### Sampling procedure and thin-section preparation for histological analysis

Cremated bone selection for histological analysis was driven by their representativeness and fragmentation degrees. 18 contexts provided material that was suitable for histological analysis. From each of the 18 cremated remains, one long bone sample was collected (humeri, n = 9; femurs, n = 9; total = 18), following the preservation, size, and color criteria outlined by Gigante et al. [19] and French et al. [52].

To ensure a blind histological assessment, each sample was labelled with a sequential alphanumeric identifier and not the original archaeological provenance label (S2 Table).

The sectioning procedure and microscopic acquisition were performed at BonesLab (University of Bologna) and BIOANTH LAB (Sapienza University of Rome), respectively. Histological and histomorphometric examination was carried out independently by two observers (AG and MG). Before the preparation of thin-section, samples larger than 5 cm were cut to obtain midshaft segments from the bone’s diaphyseal region. Following standard protocols [87,88], the fragments were embedded in a bi-component epoxy resin (EpoThin™2, Buehler Ltd.) and left to cure for 48 hours. The embedded fragments were then sectioned transversally across the midshaft using an IsoMet™ Low Speed Saw (Buehler Ltd.) with a 300 µm-thick diamond blade. The exposed surface of one of the resulting blocklets was lightly abraded using P2500 grit sandpaper (CarbiMet™, Buehler Ltd.) and polished with 1 µm polycrystalline diamond suspension (MetaDi™ Supreme, Buehler Ltd.) on a dedicated polishing cloth (TriDent™, Buehler Ltd.). After rinsing with demineralized water and air drying, the blocks were mounted on glass slides using EpoThin™ 2 epoxy resin and left to cure overnight. The mounted blocks were subsequently cut with the low-speed saw to produce ~300 µm-thick sections, which were then ground with P1200 and P2500 grit sandpaper to a final thickness of approximately 100 µm and polished again with the polycrystalline diamond suspension.

Micrographs of the thin sections were acquired at a magnification of 50x using a transmitted light microscope (Axio IMAGER.M2, Carl Zeiss Microscopy GmbH) with a 10x objective and equipped with a digital color camera for microscopy (Axiocam 807, Carl Zeiss Microscopy GmbH). Individual micrographs were automatically captured and stitched using the *Tiles tool* in ZenCore software (version 3.8; Carl Zeiss Microscopy GmbH), generating composite images of the entire section for each specimen.

### Microscopic assessment of bone preservation

The Oxford Histological Index (OHI) was applied to quantify the preservation degree and the level of microstructural degradation in each specimen [52,89,90], with scores ranging from 0 (<5% intact bone) to 5 (<95% intact bone). To ensure the reliability of the analyses, histomorphometric measurements were subsequently performed only on specimens with OHI scores between 3 and 5.

### Human vs non-human remains

Given the fragmentary nature of the selected bones, the taxon identification as human wasn’t guaranteed, and histological analysis was used to discriminate human from potential faunal remains. Human and faunal bone remains were differentiated based on specific architectural features of the non-human cortical bone, including the presence of primary plexiform vascular bone, the non-chaotic arrangement of secondary osteons, and the occurrence of osteon banding [19,53,91].

To morphometrically distinguish faunal from human remains, a threshold of 0.025 mm^2^ of osteon area (On. Ar.) was adopted [19,53,56]. All the specimens below this threshold were considered non-human.

### Age-at-death and bone microstructures in the human sub-sample

In the human sub-sample, age-at-death was estimated using the Osteon Population Density (OPD) parameter [19,92], which reflects the degree of bone remodeling associated with ageing. OPD was calculated by dividing the total number of intact and fragmented secondary osteons by the cortical surface area (Ct. Ar., mm^2^). Based on the OPD values, individuals were assigned to relative age groups, following established thresholds for human long bones [93,94]. Then, the histomorphometric results were compared with age estimations obtained through macroscopic morphological assessment. Additionally, to evaluate morphological and dimensional variations of cortical bone microstructures, 25 intact secondary osteons (On.) and their corresponding Haversian canals (Hc.) were randomly selected from each specimen. The Haversian canal area (Hc. Ar*.*, mm^2^), as well as the osteon area (On. Ar., mm^2^) and circularity index (On. Cr.) [59,60,95] were measured using ImageJ software (version 1.54p, NIH, USA) [96].

### Statistical analysis

Inter-observer error was evaluated using the technical error of measurement (TEM) analysis. For each histomorphometric parameter, %TEM indices and the R coefficient of reliability were calculated [97]. The error of measurement was considered acceptable when %TEM was lower than 5% and the R coefficient was higher than 0.75 [98,99]. For the On. Ar., Hc. Ar. and On. Cr. variables, data distribution normality was first assessed using a Shapiro-Wilk test. To ensure statistical accuracy, a non-parametric Kruskal-Wallis test, followed by a Dunn’s post-hoc test, was applied to assess differences across age groups. All statistical analyses and graphical output were performed using the R software environment (version 4.5.1) [100].

## Results

### Macroscopic analysis

The morphological analysis of the cremated remains identified a Minimum Number of Individuals (MNI) of 28 from the 26 secondary cremations analyzed (S1 Table), revealing two secondary double cremations (SU 262 and SU 339). These individuals were subsequently re-classified as SU 262A and B, and SU 339A and B, respectively.

**Fig 2:**
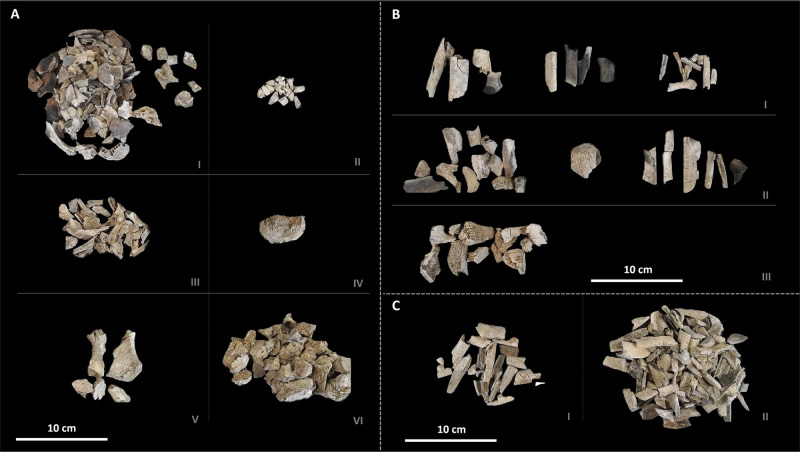
Cremated human remains from SU 262B (2008 excavation campaign) from La Cona necropolis (Teramo, 1^st^ cent. BCE-1^st^ cent. CE). Recovered elements: **(A)** Cranial and axial skeleton – I. skull; II. teeth; III. ribs; IV. scapula; V. sternum; VI. vertebrae. **(B)** Postcranial skeleton – I. humerus, radius/ulna, metacarpus; II. femur, patella, tibia/fibula; III. pelvis. **(C)** Undetermined long bone fragment – I. small diameter; II. large diameter.

As shown in Table 1, the distribution of individual weights within the examined sample displayed considerable variability, with values ranging from a minimum of 18 g for Tomb 15 to a maximum of 1.217 g for SU 262B (Fig 2). Fragment size analysis indicates a high degree of fragmentation, with most bone fragments falling within the 10–30 mm size range; only a small proportion of fragments exceeded 40 mm in maximum dimension, and no fragments larger than 60 mm were recorded.

**Table 1:** Results of morphologic and morphometric analyses of cremation burials from the La Cona necropolis.

La Cona ID	Tomb/ SU	Human sub-sample tot. weight (g)	Macroscopic assessment of age-at-death	Sex	Faunal remains	N of animal bone fragments	Odontoskeletal elements
LC2000	2	28	>20	UND	Absent	0	Absent
LC2000	5	22	>20	UND	UND	1	UND
LC2000	7	36	>20	UND	Absent	0	Absent
LC2000	12	604	>20	UND	*Ovis aries;* *Gallus gallus*	2 3	Astragalus, maxilla; Femurs, coracoid, tarsometatarsus
LC2000	13	168	20-40	UND	*Ovis* vel *capra*	1	Humerus
LC1980	14	35	>40	F	*Gallus gallus;* *Glycymeris glycymeris*	1 3	Tibiotarsus; Shell fragments
LC2000	15	18	>20	UND	Absent	0	Absent
LC2000	18	366	>20	UND	Absent	0	Absent
LC2000	19	64	>20	UND	*Ovis* vel *capra*; *Glycymeris glycymeris*; UND	1 1 Numerous	Upper molar; Mollusk shell; UND
LC1980	23	39	15-20	UND	Absent	0	Absent
LC1980	24	72	>20	UND	*Ovis aries*	3	Astragali
LC2000	25	69	>20	UND	UND	Numerous	UND
LC2000	27	341	15-20	UND	Absent	0	Absent
LC2000	28	371	10-15	UND	*Sus domesticus*	2	Maxilla and metatarsal
LC2000	*Olla*	1144	20-40	F	Absent	0	Absent
LC2011	*Olletta cineraria*	190	>20	UND	Absent	0	Absent
*La Cona*	18	134	UND	UND	Absent	0	Absent
LC2006	20	150	UND	UND	Absent	0	Absent
LC2008	262a	406	10-15	UND	Absent	0	Absent
LC2008	262b	1217	20-40	F	Absent	0	Absent
LC2008	264	190	>20	UND	Absent	0	Absent
LC2008	266	159	>20	UND	Absent	0	Absent
La Cona	267	371	>20	UND	Absent	0	Absent
*La Cona*	272	225	>20	F	Absent	0	Absent
LC2008	272	537	20-30	F	Absent	0	Absent
*La Cona*	337	535	>40	F	Absent	0	Absent
*La Cona*	339a	280	>40	UND	*Ovis* vel *capra*	1	Lower molar
*La Cona*	339b	776	>40	F	*Ovis* vel *capra*	2	Femur and tibia

LC = *La Cona* (abbreviation according to the different excavation campaigns:1980; 2000; 2006; 2008; 2011); *olla* = urn (as reported in the excavation records); *olletta cineraria* = small cinerary urn (as reported in the excavation records); UND = undetermined; F = female. Age-at-death estimations are expressed in years.

All adult individuals exhibited the greatest degree of underrepresentation of the skeletal elements. In these cases, the remains consisted almost exclusively of cranial fragments and long bones (S2 Table). By contrast, the subadult individuals (except for the subject identified in Tomb 23, whose total weight was calculated at 39 g) had an overall weight greater than the average weight distribution within our sample (305 g), ranging between 341 g and 406 g.

Morphological age-at-death and sex estimations based on the morphological and morphometric analysis of the La Cona cremated remains are summarized in Table 1. Most skeletal and dental fragments exhibit a white-calcined structure and a whitish to greyish coloration, while only a few fragments display a broader range of color alterations, including blue-grey, black-grey, and black-brownish hues (S3 Table).The chromatic characteristics of the cremated remains suggest exposure to relatively high-temperature burning conditions, as indicated by the prevalence of light grey to white coloration across the assemblage. Experimental and archaeological studies have shown that such colours are generally associated with advanced oxidation of organic components and are commonly observed in bones burned at temperatures typically exceeding 600–700 °C [77]. However, colour alone cannot be considered a precise or unambiguous proxy for pyre temperature, as it is also influenced by factors such as burning duration, oxygen availability, body position on the pyre, and post-depositional alteration [25,32,77].

Morphological age-at-death could be estimated for 26 of the 28 individuals, as two funerary contexts (SU 18; SU 20) were excluded due to the extreme fragmentation of the remains. The remaining individuals were grouped into five age classes according to Buikstra & Ubelaker [69]: 2 subadults (10–15 years), 3 adolescents (15–20 years), 3 adults (20–40 years), 1 young adult (20–30 years), and 3 adults (20–40 years), 5 mature adults (>40 years), and 12 adults (>20 years).

Biological sex estimation based on morphological features classified seven individuals as females, and all belonged to the adult and mature adult age classes. Four additional individuals (Tomb 14, *Olla*, SU 337, and SU 339B) were classified as probable females. While this proportion may appear relatively high for a cremated assemblage, it should be interpreted cautiously. Sex assessment was based exclusively on the preservation of a limited number of morphologically diagnostic traits, primarily derived from pelvic and cranial fragments, and no sex estimation was attempted when such traits were ambiguous or poorly preserved. The observed predominance of female classifications may therefore reflect preservation bias and differential survival of sexually dimorphic traits, rather than a true demographic pattern within the La Cona assemblage. Accordingly, sex estimates are treated as tentative and are not used as a basis for broader demographic or social interpretation.

The morphological examination revealed the presence of an ivory worked element in Tomb 19 (S1 Fig), as well as faunal remains (S2 Fig) mixed with the human osteological material in ten depositions (Tomb 5, Tomb 12, Tomb 13, Tomb 14, Tomb 18, Tomb 19, Tomb 24, Tomb 25, Tomb 28, and SU 339). Zooarchaeological analysis identified caprine (*Ovis* vel *capra;* in only a few instances specifically identified as sheep, *Ovis aries*), pigs (*Sus domesticus*), poultry (*Gallus gallus*), and mollusks (*Glycymeris glycymeris*). Both cranial and postcranial elements were present among domestic mammal remains. In Tomb 28, the pig remains belong to a subadult individual. No precise age-at-death data are available for the caprine; however, their size and morphology suggest subadult or adult individuals rather than juveniles. Among the remains of *Gallus gallus*, limb elements from mature individuals were identified. The three astragali of *Ovis aries* discovered in Tomb 24 exhibit indications of processing, and their provenance could be traced to three distinct animals. Numerous unidentified bone fragments were recorded in Tomb 19 and Tomb 25. Although listed in Table 1, they were not individually counted but were noted as quite abundant. The faunal remains exhibit a color pattern consistent with the human ones, except for burials SU 339 and Tomb 13, where a slightly different chromatic pattern was observed.

### Microscopic analysis

**Fig 3:**
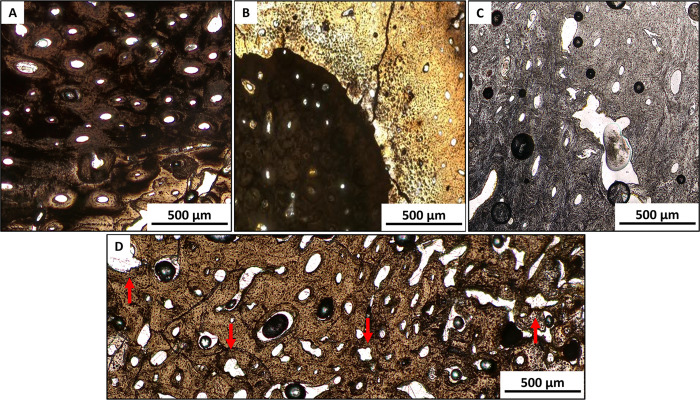
Chromatic and morphological alterations resulting from the cremation process. **(A)** LC02, humerus – carbon inclusions in bone matrix; **(B)** LC14, femur – chromatic alterations between periosteum and endosteum; **(C)** LC07, femur calcined bone with heat-induced microfractures; **(D)** LC12, femur – osteon splitting phenomenon.

Qualitative histological analysis confirmed that the chromatic changes and microfractures observed microscopically were consistent with alteration patterns identified in the macroscopic assessment (Fig 3 and S4 Table). For example, under microscopic examination, osteological specimens exhibiting blackish-brown staining showed substantial carbon inclusions in periosteal and midcortical regions (Fig 3A). The specimen LC14 exhibited, at a histological level, chromatic alterations between the periosteum and the endosteum, corroborating previous macroscopic observations (Fig 3B). White-calcined and/or white-gypsum remains (LC07 and LC17) displayed characteristic calcination effects [101] in thin section, including bone matrix discoloration, micro-fractures, and progressive obliteration of osteocytic lacunae (Fig 3C). The “osteon splitting phenomenon” (*i.e.,* the transverse cracking emanating from the Haversian canals and osteons due to heating [102]) was also identified throughout the entire sample (Fig 3D).

**Fig 4:**
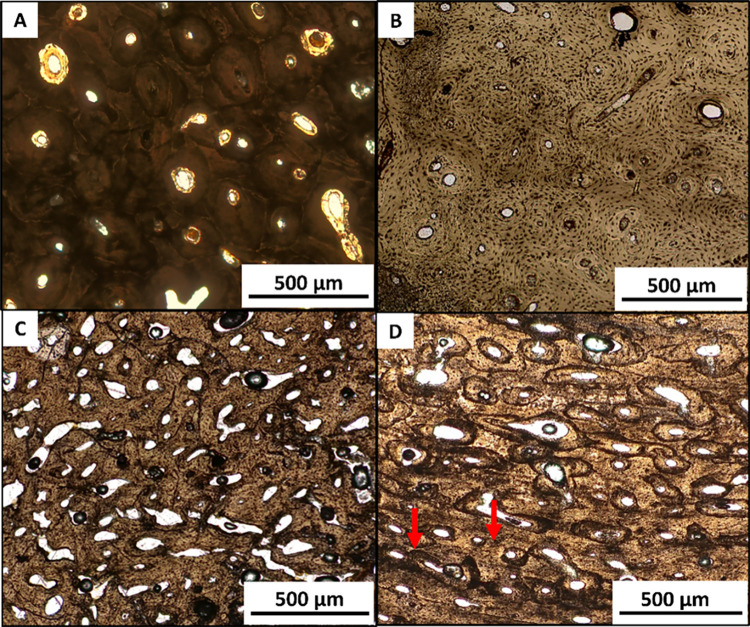
Microscopic features of the Haversian bone system. **(A)** LC14, femur – secondary osteons in adult bone; **(B)** LC08, femur – Haversian system in immature bone; **(C)** LC12, femur – mature bone with resorption gaps; **(D)** LC11, humerus – red arrows indicate osteon banding.

The arrangement of bone microstructures indicates that the examined specimens exhibited the typical pattern of the human Haversian system, characterized by the presence of both intact and fragmentary secondary osteons chaotically arranged. The observed degree of bone remodeling varied according to age [92] (Fig 4A and 4B). Only two specimens (LC07; LC08) showed sparse osteons within the bone matrix, with a predominance of primary osteons — *i.e.*, with poorly defined cement line [103] — over secondary osteons (Fig 4B). Moreover, in LC08, lamellar bone was observed at the periosteal surface. The presence of primary osteons and lamellar bone in human samples has been associated with immature cortical bone undergoing further development [103]. Conversely, LC12 exhibited resorption gaps typical of mature cortical bone tissue (Fig 4C). The qualitative histological analysis of sample LC11 revealed the presence of osteon banding, characterized by the alignment of osteons in parallel bands [91] (Fig 4D).

All the histomorphometric measurements are reported in S5 Table.

Table 2 summarizes the histomorphometric results, reporting mean secondary osteon areas (On. Ar.), mean Haversian canal areas (Hc. Ar.), and mean osteon circularity index (On. Cr.) with standard deviations (SD).

**Table 2:** Results of the histomorphometric analysis of cremated cortical bones.

SpecimenID	Mean On. Ar. (mm^2^)	SD	Mean Hc. Ar. (mm^2^)	SD	Mean On. Cr.	SD	Macroscopic analysis	Qualitative histology	Histomorphometry
LC01	0.012	0.005	0.002	0.001	0.953	0.027	H	H	NH
LC02	0.025	0.011	0.003	0.004	0.892	0.029	H	H	H
LC03	0.032	0.008	0.003	0.002	0.918	0.025	H	H	H
LC04	0.036	0.011	0.007	0.007	0.884	0.044	NH?	H	H
LC05	0.026	0.015	0.003	0.002	0.908	0.044	H	H	H
LC06	0.027	0.007	0.004	0.003	0.906	0.038	H	H	H
LC07	0.029	0.009	0.002	0.001	0.899	0.038	H	H	H
LC08	0.035	0.014	0.004	0.003	0.922	0.027	NH?	H	H
LC09	0.025	0.012	0.003	0.002	0.907	0.035	NH?	H	H
LC10	0.030	0.009	0.003	0.002	0.848	0.040	H	H	H
LC11	0.025	0.012	0.003	0.002	0.881	0.034	H	H	H
LC12	0.026	0.007	0.003	0.002	0.764	0.072	NH?	H	H
LC13	0.025	0.005	0.003	0.002	0.871	0.045	H	H	H
LC14	0.037	0.014	0.003	0.001	0.875	0.050	H	H	H
LC15	0.028	0.013	0.003	0.002	0.945	0.020	H	H	H
LC16	0.027	0.012	0.003	0.001	0.925	0.023	H	H	H
LC17	0.028	0.008	0.004	0.002	0.898	0.048	NH?	H	H
LC18	0.032	0.018	0.003	0.002	0.910	0.050	H	H	H

*On. Ar* = secondary osteons area (mm^2^); *Hc. Ar.* = Haversian canal area (mm^2^);*On. Cr.* = osteon circularity index; SD = standard deviation; *H* = human; *NH* = non-human.

In addition, for each specimen, taxonomic determination is reported according to macroscopic and microscopic observations. The reliability of the measurements was established since %TEM and R values fell within the expected threshold (On. Ar. %TEM = 2.65, R = 0.998; Hc. Ar. %TEM = 2.61, R = 0.998; On. Cr %TEM = 1.44, R = 0.903).

One specimen (LC01), although morphologically and histologically identified as human, exhibited a mean On. Ar. value below the established human threshold of 0.025 mm^2^ [19,53–57]. Consequently, it was classified as faunal remains and excluded from the OPD analysis.

Table 3 presents the cortical surface area (Ct. Ar., mm^2^), the number of intact and fragmentary secondary osteons, and the resulting OPD value for each specimen. Comparison with reference OPD values [82,83] allowed the classification of 13 adults (20–40 years; OPD range = 11.83–28.59; mean OPD = 19.60; SD = 6.77), one mature individual (> 40 years; OPD = 33.13), a young child (2–7.9 years; OPD = 1.23), an older child (8–12.9 years; OPD = 3.46), and an adolescent (13–18 years; OPD = 7.68). In addition, for OPD, the reliability of the measurements was established (%TEM = 2.06, R = 0.998). Histological age-at-death and previous macroscopic estimations are compared in Table 3.

**Table 3:** Results of Osteon Population Density (OPD) for histological age at death estimation.

Specimen ID	Bone sample	Ct. Ar. (mm^2^)	OPD (sd)	Histological age classes*	Morphological age classes
LC02	humerus	52.98	27.76 (0.02)	20-40	20-40
LC03	humerus	36.29	21.71 (0.16)	20-40	>40
LC04	femur	34.48	11.83 (0.06)	20-40	>40
LC05	humerus	42.97	13.74 (0.18)	20-40	>20
LC06	humerus	25.60	17.58 (0.03)	20-40	>20
LC07	femur	64.76	3.46 (0.02)	8-12.9	15-20
LC08	femur	99.30	1.23 (0.01)	2-7.9	10-15
LC09	femur	32.12	21.72 (0.13)	20-40	20-40
LC10	humerus	22.04	19.88 (0.50)	20-40	>20
LC11	humerus	53.48	28.59 (0.14)	20-40	UND
LC12	femur	52.14	33.13 (0.11)	>40	UND
LC13	humerus	54.19	26.26 (0.27)	20-40	20-40
LC14	femur	63.43	17.36 (0.12)	20-40	>20
LC15	femur	45.96	26.28 (0.83)	20-40	>20
LC16	humerus	29.30	20.55 (0.08)	20-40	>20
LC17	humerus	19.15	7.68 (0.41)	13-18	20-30
LC18	femur	42.67	23.95 (0.02)	20-40	>40

Age classes reference for infants and children from (Pitfield *et al*., [83]); age classes reference for adults from (Miskiewicz *et al.,* [94]); *Ct. Ar.* = cortical area (mm^2^); *UND* = undetermined.

It is noteworthy that microscopic analysis enabled age estimation for two individuals (LC11; LC12) who could not be determined on a morphological basis.

**Fig 5:**
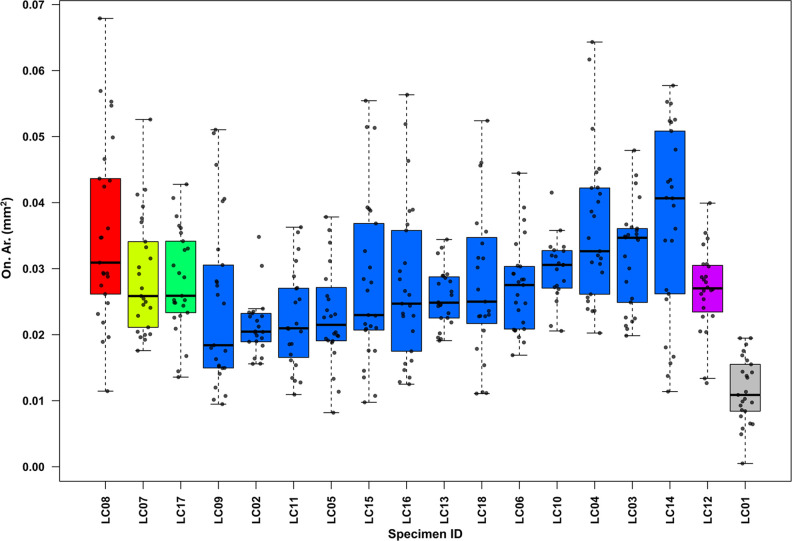
Box and whisker plots of secondary osteons area (On. Ar.) in the analyzed sample. Boxplots are grouped from the youngest to the oldest individuals according to microscopic age-at-death assessment. Colors indicate different age classes (red = young child, yellow = older child, green = adolescent, blue = adults, purple = mature adult, grey = faunal); within each group, specimens are ordered by increasing median values. Each bullet point represents an individual measurement; outliers were excluded. The faunal specimen (LC01) is highlighted in grey.

**Fig 6:**
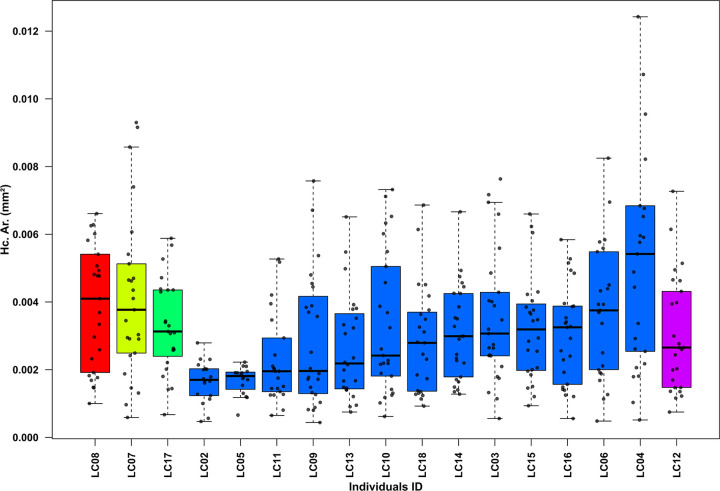
Box and whisker plots of Haversian canal area (Hc. Ar.) in the human sub-sample. Boxplots are grouped from the youngest to the oldest individuals according to microscopic age-at-death assessment. Colors indicate different age classes (red = young child, yellow = older child, green = adolescent, blue = adults, purple = mature adult); within each group, specimens are ordered by increasing median values. Each bullet point represents an individual measurement; outliers were excluded.

As illustrated in Figs 5 and 6, both On. Ar. and Hc. Ar. show marked inter-individual variability, following comparable age-related trends. The youngest individual (LC08, classified as a young child by OPD) exhibited relatively high mean values (On. Ar. = 0.035 mm^2^; Hc. Ar. = 0.004 mm^2^) and a higher range of variability within the entire sample (min On. Ar. = 0.011 mm^2^; max On. Ar. = 0.067 mm^2^). The older child (LC07) and the adolescent (LC17) displayed similar or slightly lower mean values (On. Ar. = 0.029 and 0.028 mm^2^; Hc. Ar. = 0.002 and 0.004 mm^2^, respectively) and reduced variability compared to LC08. Among the 13 adults (20–40 years), On. Ar. ranged from 0.025 (LC09) to 0.037 mm^2^ (LC14), while Hc. Ar. ranged from 0.003 (LC02) to 0.007 mm^2^ (LC04). The oldest individual based on microscopic assessment (LC12, > 40 years) reports comparatively lower mean values (On. Ar. = 0.026 mm^2^; Hc. Ar. = 0.002 mm^2^).

LC01, identified as non-human, showed the lowest mean On. Ar. in the entire sample (On. Ar. = 0.012 mm^2^). Due to the uncertain taxonomic attribution, it was decided to exclude LC01 from histological age-at-death estimation.

Statistical analyses confirmed significant differences among the five age groups for both parameters. For On. Ar., the Kruskal-Wallis test (χ² = 38.16, df = 4, p < 0.01) and Dunn’s Honest Significant Difference test revealed significant differences between adults and the older child, as well as between the young child and the mature adult. Similarly, Hc. Ar. values were significantly different among groups (Kruskal-Wallis: χ² = 14.43, df = 4, p = 0.006), with Dunn’s post-hoc indicating significant differences between adults, young child, older child, and adolescent; significant differences were also found between the young and older child.

**Fig 7:**
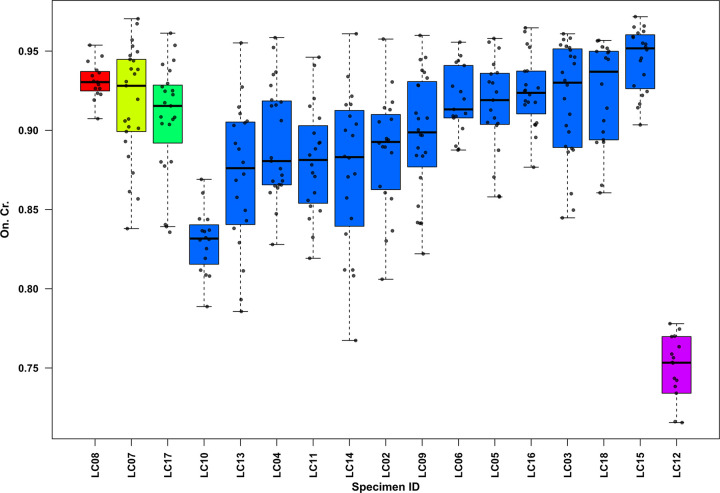
Box and whisker plots of osteon circularity (On. Cr.) for the human sub-sample. Boxplots are grouped from the youngest to the oldest individuals according to microscopic age-at-death assessment. Colors indicate different age classes (red = young child, yellow = older child, green = adolescent, blue = adults, purple = mature adult); within each group, specimens are ordered by increasing median values. Each bullet point represents an individual measurement; outliers were excluded.

The distribution of osteon circularity (On. Cr.) for the human sub-sample (N = 17) is shown in Fig 7. Box and whisker plots illustrate intra-individual variability. High mean On. Cr. values are recorded in the younger individuals (LC08 = 0.899; LC07 = 0.922, and LC17 = 0.898). Adults show values ranging from 0.848 (LC10) to 0.945 (LC15); the mature individual (LC12) exhibited the lowest mean On. Cr. in the entire human sub-sample (0.764), thus identifying it as a probable outlier. Differences in On. Cr. values are statistically significant (Kruskal-Wallis rank sum test: χ² = 77.46, df = 4, p-value < 0.01). Dunn’s post-hoc test also indicates significant differences between all age classes.

## Discussion

By integrating macroscopic and histological analyses, this study can offer a complementary approach to the recent multi-analytical protocol proposed by Waltenberger et al*.* [104] for ancient cremations, which currently does not incorporate histological examinations.

At La Cona necropolis, the predominance of single secondary cremations closely mirrors the funerary patterns observed in other Late Republican and Imperial Roman cemeteries in northern [105–108] and central Italy [109–113]. This correspondence suggests that the funerary practices most commonly employed across the Italian peninsula were also adopted at La Cona.

In adult tombs, the majority of recorded weights (<305 g) suggest a general underrepresentation of skeletal elements compared to the expected values for archaeological cremated remains. Expected weights generally range between 1001.5 g and 2422.5 g, with an average of 1625.9 g [29]. These low weights, along with a lack of specific anatomical districts(*i.e.*, facial bones, axis, pelvis, hands, and feet) may suggest a specific ritual act involving the burnt remains, known as *ossilegium*, a practice favoring the selective collection of cranial and long bones [19,114,115]. These specific bone fragments have been frequently found at the La Cona necropolis.

Interestingly, subadults have weights above the average for the entire sample and a more heterogeneous anatomical representation. This result can be attributed to a *pars pro toto* collection strategy [30]. These distinct practices likely reflect ritual behaviors differing by age class.

The analysis of the heat-induced alterations revealed no significant differences between human and faunal remains, suggesting that animals — or portions of them — were burnt with or in the same way as the human bodies, possibly as food offerings or symbolic companions in the afterlife [116]. However, macroscopic colour alone cannot be considered a reliable or exclusive indicator of burning temperature or pyre conditions. Alternative explanations, including post-cremation depositional practices involving the association of human and animal remains, cannot be excluded and should be considered when interpreting colour similarities within the assemblage [74].

The presence of smoothed, burnt astragali of *Ovis aries* in Tomb 24 (S2 Fig), commonly associated with juvenile burials in Roman funerary contexts, but also found in adult tombs [117], suggests multiple symbolic meanings. Similar finds in Italic and Greek funerary contexts—including the necropolis of Locri Epizefiri (southern Italy), where hundreds of astragali were arranged in deliberate, often apotropaic patterns—underscore their ritual importance [118]. A comparable case is tomb 101 at Varranone (Poggio Picenze, central Italy), where an adult woman was buried with over a hundred caprine astragali (*Ovis aries* and *Ovis* vel *capra*) laid in a festoon-like pattern along her left side, interspersed with small clusters of iron nails. None of the bones showed signs of modification, reinforcing an interpretation centered on magical protection—possibly intended to safeguard the deceased or to ward off their return [119]. By contrast, in the so-called “*Tomba Zeta*” of an 8–9-year-old boy at Populonia, around one hundred astragali—many smoothed or bearing cut marks—likely evoke the child’s play habits, intended to accompany him into the afterlife [108]. These examples reflect the semantic variability of astragali in funerary contexts, shaped by age, modification, and spatial arrangement. Generally, the significance of these items may vary depending on the age of the deceased and the nature of the bone modifications, which can represent toys for children or amulets for adults, and accompany the individual on their final journey.

An ivory fragment from Tomb 19 (S1 Fig) can be attributed to decorative elements of a funeral bed. The use of funeral beds in cremation rituals at the Imperial Roman necropolis of La Cona has already been demonstrated during past excavation campaigns [70], as well as being attested in other necropolises of the Empire [113].

Animal remains, primarily caprine, followed by *Gallus gallus* and mollusks, were recovered from various burials without a discernible pattern of preference. Pig bones were found exclusively in Tomb 28, associated with a juvenile individual, possibly reflecting a specific but uncertain ritual significance.

Thin sections from seven specimens showed carbon inclusions, visible as well-defined, black areas in the periosteal and midcortical regions, around the Haversian canals (Fig 3A and 3B). This phenomenon has also been documented by Hanson et al. [101] and Lemmers et al. [102]. Furthermore, these specimens exhibited enlarged and darkened osteocyte lacunae, a feature frequently observed in cremated remains in conjunction with carbon inclusions [102,120]. Sometimes, these inclusions can be lost with increasing temperature [102] or unstable pyre conditions [121] Recent studies indicate that carbon and oxygen isotopic signals, and thus coloration, can vary between the periosteal and endosteal regions of bone fragments [121]. These differences may be linked to uncontrolled burning conditions such as oxygen availability, atmospheric pressure, and the season of cremation [121].In the La Cona sample, color differences were observed between the endosteum and periosteum in the LC07 and LC08 specimens, both of which were identified as non-adult individuals. This variation may be related to low bone density and body mass in non-adults, as well as differences in bone turnover that could accelerate carbon exchange [121,122].

Despite these specimens receiving a relatively low OHI score (S4 Table), due to moderate carbon inclusions, the bone microstructures were not completely hidden, thus allowing histomorphometric analysis. A greyish-white, calcined-white, or chalky-white coloration of the cortical surface, a symptom of calcinated bone, can obliterate the bone microstructures. Hanson et al*.*[101] traced such effects to very high temperatures (>900 °C), consistent with bioapatite crystallites rearrangement [123]. In addition, heat-induced microfractures found in most of the specimens are comparable to those described by Cambra-Moo et al. [41] in cremated human remains from the Lagunita I site (Spain, 1^st^ millennium BCE), as well as on forensic samples from Hummel [38] and Squires et al. [123]. Notwithstanding the documented cases of bone matrix discoloration, thermal alterations, and osteon splitting in the white-calcined specimens from La Cona necropolis (Fig 3C and 3D), it is clear that bone microstructures appear to be preserved. Consequently, it can be hypothesized that the funeral pyre could have reached elevated temperature, to completely destroy the bone microfeatures. Several studies on cremations have shown that a significant amount of cortical microstructures can retain their shape and remain visible for analysis, even at temperatures exceeding 1000 °C [41,46,58,124,125].Qualitative histology was conducted to investigate the arrangement patterns of secondary osteons within the cortex, revealing a typical human Haversian system in all specimens. Human thin sections displayed chaotically arranged secondary osteons, which also reflect the age-related remodeling [53,92]. Interestingly, one specimen (LC01, Tomb 2) was estimated as possibly human from qualitative histology, but as non-human from histomorphometry (mean On. Ar. < 0.025 mm^2^) [19,53–57]. LC08 (Tomb 28)’s femoral portion showed sparse secondary osteons and almost no remodeling (Fig 4B), with primary osteons found in the entire cortical surface and periosteal lamellar bone. This pattern is typical of immature human bone [47,126], consistent with its low OPD value (1.39). The combination of these data confirmed LC08 as a young child (2–7.9 years) [94].

Conversely, LC12 (SU 20)’s femur thin section exhibited intense resorption lacunae across the cortical surface, irregular osteons, and prominent Haversian canals. This histological pattern is consistent with a mature human bone (Fig 4C; Fig 6, p. 198 in Grosskopf [127]). This hypothesis is also supported by its high OPD value (mean OPD = 33.89).

In addition, histological analysis identified the phenomenon of osteon banding in a human humerus fragment (LC11, SU 18) (Fig 4D). The presence of lamellar bone with osteons aligned in parallel rows is usually employed in the discrimination of human vs. non-human bone in cremated or commingled remains, as it is attributed to large herbivores [19,52,53]. Nonetheless, Andronowski et al. [91] proposed that the presence of osteon bands in a single specimen may not necessarily be diagnostic of non-human bone. Indeed, as demonstrated by Mulhern [47], there are some documented cases of osteon banding in human bones. However, the underlying cause of the presence of these bands in humans remains unclear to date. It is not yet established whether this phenomenon may be influenced by the occurrence of mechanical stress [91] or other factors.

Osteon Population Density (OPD), a method previously used mainly in forensic science [40,58], was here applied to cremated archaeological remains, similar to the approach in Gigante et al. [19] for the Tomb of Nestor’s Cup found in the Pithekoussai necropolis (Ischia, 8^th^ century BCE). As outlined in Gigante et al. [19], although thin sections were obtained from different long bones (*i.e.*, humeri and femurs), this did not affect the histomorphometric analysis, as no significant micro-anatomical differences were observed between these skeletal elements.

It’s worth noting that bone shrinkage due to the cremation process (estimated to range between 10 and 30% in human bones [128]) could affect histomorphometric analyses, but it cannot be easily estimated in archaeological samples. Anyhow, assuming a reasonably similar bone shrinkage rate for all anatomical segments where histology could be read, the present OPD estimates can be used as a proxy for the age-at-death distribution within the sample, rather than providing accurate estimates of true age at death. Consequently, the ages-at-death are presented here adopting very broad age ranges.

The reliability of the histological approach to the age at death was demonstrated, with more than 60% agreement between the two methods. It is also noteworthy that histomorphometric analysis facilitated the estimation of the age at death for two individuals (LC11 and LC12) who would otherwise have remained undeterminable. Nevertheless, discrepancies between the two methods were observed. In six of the seventeen cases analyzed (LC03; LC04; LC07; LC08; LC17; LC18), the age estimates derived from OPD were found to be lower than those obtained from macroscopic analysis. This underestimation could be attributed to the partial loss of some bone microstructures during the cremation process [40,123]. The age-at-death estimation has revealed that children and adolescents were equally represented in the burial space at the Imperial Roman necropolis of La Cona. The absence of infants under 2 years of age may reflect dedicated funerary rituals for this age group, as also documented in the coeval Paduan necropolises [105,106]and at Egnazia in southern Italy [129]. During the protohistoric phase of ^th^e La Cona necropolis (9^th^–3^rd^ century BCE), infants and perinatal children were buried alongside other members of the community [73]. However, with the transition from the Iron Age to the Roman period, these youngest individuals appear to have been excluded from adult burial grounds, a well-documented practice in Roman contexts [130,131]. According to Pliny the Elder, infants without teeth were not cremated but instead inhumed [132]. However, exceptions can be found. For example, at the Roman cremation necropolis of Bologna, perinatal and infant individuals were buried in the same area as adults [133].

Microscopic estimation of age-at-death revealed age-related histomorphometric trends. For example, both mean On. Ar. and mean Hc. Ar*.* tend to be lower in the majority of the adult specimens (76%) than the younger ones, while the mature individual showed lower mean areas, with significant differences confirmed by Kruskal-Wallis and Dunn’s post-hoc tests. These data align with previous studies [59,61–66]. According to Gibson et al. [67] and O’Brien et al. [68], the age-related reduction in osteon size may reflect increased bone density and compact cement lines, which reduce the risk of microfracture propagation. Similarly, Britz et al. [66] hypothesize that the presence of smaller osteons attenuates the expansion of resorption gaps and prevents excessive weakening in mature bone matrix. These trends have also been observed in ribs [53,60], but not in metacarpals [69].

Early analyses of On. Cr. suggested a more rounded shape in the osteons of mature individuals, regardless of sex [59], consistently with later interpretations relating increased circularity to bone ageing and mechanical adaptation [66–68,134–136]. However, no standardized method for analyzing this parameter currently exists [137]. The mean On. Cr. parameters recorded in the humeri and femurs from La Cona (Table 2) fell within the ranges of human variability reported in the literature [51,136,138]. However, a clear correlation between higher osteon circularity and advanced age could not be established in this sample, as some adult individuals (Fig 7) exhibited lower indices than younger subjects. For example, the mature individual (LC12, SU 20) showed the lowest mean On. Cr. (0.764) in the entire sample. Leiss et al*.* [139] have reported similar findings, describing less circular, elliptical osteons in mature bone. This condition may be attributed to the imperfect transverse sectioning that can artificially alter osteons’ shape [137].

## Conclusions

By combining macro- and microscopic analyses, this study shed light on the demographic profile of La Cona’s cremated skeletal series during the Imperial Roman period.

The use of the Osteon Population Density (OPD) parameter proved particularly effective, allowing for relative age-at-death estimations within the examined sample, even in cases where traditional morphological methods were limited by fragmentation or poor preservation.

In addition, this research represents the first documentation, in Imperial Roman cremated skeletal series, of osteon shape and size variation through histological age, demonstrating that microscopic bone structures can retain significant informative potential despite thermal alteration. Microstructural parameters, especially secondary osteon areas, proved useful for distinguishing between human and animal specimens, allowing the correct diagnosis for cases that were not macroscopically evident.

Beyond the methodological contribution, this work sheds light on the ritual complexity and symbolic depth of Roman cremation practices at La Cona, where age-dependent patterns of post-cremation bone selection in *ossilegium* and the inclusion of faunal remains reflected differentiated funerary rituals, dependent on age-at-death. These findings demonstrate that, while not perfect, histology is a reliable tool when used alongside macroscopic analysis in the investigation of cremated human remains. Furthermore, the histological and histomorphometric framework presented in this study could be proposed as an additional methodology for multi-analytical approaches currently used for ancient burnt bones.

### Additional information

The skeletal and dental collection from La Cona is currently housed at the Archaeology Laboratories of the Department of Cultural Heritage, University of Padua, Padua, Italy; the identifiers of the examined specimens are provided in Table 1 of the manuscript, and access and all necessary permits were issued by the Soprintendenza Archeologia, Belle Arti e Paesaggio per le province di L’Aquila e Teramo for the described study, which complied with all relevant regulations.

## Supporting information

S1 Table Excavation details of funerary contexts from La Cona necropolis (Teramo, Abruzzo, 1^st^ cent. BCE – 1^st^ cent. CE). (DOCX)

S2 Table Number of skeletal elements and total weight recorded for each individual. (XLSX)

S3 Table List of funerary contexts selected for histological analysis, blind histological ID, sampled bone, and chromatic alteration. (DOCX)

S4 Table Observed characteristics to determine bone preservation according to Oxford Histological Index (OHI). (DOCX)

S5 Table Hiistomorphometric data and osteon count for aeach analysed parameter in the La Cona sample (On. Ar.; Hc. Ar.; On. Cr.; OPD). (XLSX)

S1 Fig Ivory elements from La Cona necropolis (Teramo, 1^st^ cent. BCE – 1^st^ cent. CE). Fragment of a decorative ivory element from Tomb 19. (TIF)

S2 Fig Burned faunal remains from La Cona necropolis (Teramo, 1^st^ cent. BCE – 1^st^ cent. CE). (A) Limb elements of *Gallus gallus* from Tomb 12; (B) Smoothed astragali of *Ovis aries* from Tomb 24. (TIF)
