# Supplementary material for: Macroscopic and histological analyses of cremated remains from the Imperial Roman necropolis of La Cona (1st cent. BCE-1st cent. CE, Teramo, Italy)
Source: PLoS One. 2026 Apr 22;21(4):e0345498. doi: 10.1371/journal.pone.0345498 (PMC13102198; doi:10.1371/journal.pone.0345498)
Supplement: S1 Table — (DOCX) [file pone.0345498.s001.docx]

S1 Table. Excavation details of funerary contexts from La Cona necropolis (Teramo, Abruzzo, 1^st^ cent. BCE – 1^st^ cent. CE).

| **Site ID** | **Year of excavation** | **Tomb/ SU** |
| --- | --- | --- |
| LC2000 | 2000 | 7 |
| LC2000 | 2000 | 5 |
| LC2000 | 2000 | 25 |
| LC2000 | 2000 | 2 |
| LC2000 | 2000 | 15 |
| LC2000 | 2000 | 19 |
| LC1980 | 1980 | 24 |
| LC2000 | 2000 | 12 |
| La Cona | - | 267 |
| LC2011 | 2011 | *olletta cineraria* |
| LC2008 | 2008 | 266 |
| LC2008 | 2008 | 264 |
| La Cona | - | 272 |
| LC2000 | 2000 | 18 |
| LC1980 | 1980 | 14 |
| La Cona | - | 337 |
| La Cona | - | 339 |
| LC2008 | 2008 | 262 |
| LC2000 | 2000 | 28 |
| LC2000 | 2000 | 27 |
| LC1980 | 1980 | 23 |
| LC2008 | 2008 | 272 |
| LC2000 | 2000 | 13 |
| LC2000 | 2000 | *olla* |
| La Cona | - | 18 |
| LC2006 | 2006 | 20 |

*SU = Stratigraphic Unit.*
