# Supplementary material for: Macroscopic and histological analyses of cremated remains from the Imperial Roman necropolis of La Cona (1st cent. BCE-1st cent. CE, Teramo, Italy)
Source: PLoS One. 2026 Apr 22;21(4):e0345498. doi: 10.1371/journal.pone.0345498 (PMC13102198; doi:10.1371/journal.pone.0345498)
Supplement: S3 Table — (DOCX) [file pone.0345498.s003.docx]

S3 Table. List of funerary contexts selected for histological analysis, blind histological ID, sampled bone, and chromatic alteration.

| **Tomb/SU** | **Histological ID** | **Bone** | **Color** |
| --- | --- | --- | --- |
| T 2-sett. 1 | LC01 | femur | greyish-white |
| T 13 | LC02 | humerus | greyish-white |
| T 14 | LC03 | humerus | black-brownish |
| T 18 | LC04 | femur | brownish |
| T 19 | LC05 | humerus | greyish |
| T 24 | LC06 | humerus | greyish |
| T 27 | LC07 | femur | greyish-white |
| T 28 | LC08 | femur | brownish |
| *olla* | LC09 | femur | black-brownish |
| *olletta cineraria* | LC10 | humerus | greyish-white |
| SU 18 | LC11 | humerus | brownish |
| SU 20 | LC12 | femur | brownish |
| SU 262B | LC13 | humerus | greyish-white |
| SU 266 | LC14 | femur | greyish-white |
| SU 267 | LC15 | femur | greyish-white |
| SU 272 | LC16 | humerus | black-brownish |
| LC 2008 SU 272 | LC17 | humerus | greyish-white |
| SU 339B | LC18 | femur | greyish-white |

*T = Tomb; SU = Stratigraphic Unit.*
