# Supplementary material for: Macroscopic and histological analyses of cremated remains from the Imperial Roman necropolis of La Cona (1st cent. BCE-1st cent. CE, Teramo, Italy)
Source: PLoS One. 2026 Apr 22;21(4):e0345498. doi: 10.1371/journal.pone.0345498 (PMC13102198; doi:10.1371/journal.pone.0345498)
Supplement: S4 Table — (DOCX) [file pone.0345498.s004.docx]

S4 Table. Observed characteristics to determine bone preservation according to Oxford Histological Index (OHI) [1].

| **Histological ID** | | **Average OHI value** | | **Description** | | **Notes** | |
| --- | --- | --- | --- | --- | --- | --- | --- |
| LC01 | | 3,4 | | Macroscopically, it appears greyish-white throughout the section. Microscopic appearance is tan to dark brown. Secondary Haversian system, lamellar bone, and lacunae are visible in the majority of the thin section. Carbon deposition in periosteal and midcortical. Large cracks are present. | | No grinding scratches. | |
| LC02 | | 3,8 | | Macroscopically, it appears greyish-white throughout the section. Microscopic appearance is tan to brown. Secondary Haversian system, lamellar bone, and lacunae are visible in the majority of the thin section. Carbon deposition in periosteal and midcortical. Large and microcracks are present, along with the osteon splitting phenomenon. | | No grinding scratches. | |
| LC03 | | 3,1 | | Macroscopically, it appears black-brownish throughout the section. Microscopic appearance is grey. Secondary Haversian system, lamellar bone, and lacunae are visible in the majority of the thin section. Carbon deposition in periosteal and midcortical, especially in lacunae. Large cracks and  the osteon splitting phenomenon are present. | | No grinding scratches. | |
| LC04 | | 3,3 | | Macroscopically, it appears brownish throughout the section. Microscopic appearance is dark brown. Secondary Haversian system, lamellar bone, and lacunae are visible in the majority of the thin section. Carbon deposition in periosteal and midcortical. Large cracks and the osteon splitting  phenomenon are present. | | Presence of grinding scratches. | |
| LC05 | | 4 | | Macroscopically, it appears greyish throughout the section. Microscopic appearance is dark brown. Secondary Haversian system, lamellar bone, and lacunae are visible in the majority of the thin section. Carbon deposition in periosteal and midcortical. | | No grinding scratches. | |
| LC06 | | 4,2 | | Macroscopically, it appears greyish throughout the section. Microscopic appearance is brown. Secondary Haversian system, lamellar bone, and lacunae are visible in the majority of the thin section. Light carbon deposition in periosteal. Large cracks and  the osteon splitting phenomenon are present. | | No grinding scratches. | |
| LC07 | | 3,1 | | Macroscopically, it appears greyish-white throughout the section. Microscopic appearance is grey.  Secondary Haversian system, Haversian system, and lamellar bone are visible in the majority of the thin sections. Lacunae are partially obliterated. No carbon deposition. Large cracks and the osteon splitting phenomenon are present. | | Presence of grinding scratches. | |
| LC08 | | 4,5 | | Macroscopically presents the so-called sandwich effect. Microscopic appearance is tan in the periosteal to brown in the midcortical. Secondary Haversian system, lamellar bone, and lacunae are visible in the majority of the thin section. Carbon  deposition in midcortical. Microcracks are present. | | Presence of grinding scratches. | |
| LC09 | | 4,3 | | Macroscopically, it appears dark brown throughout the section. The microscopic appearance is brown. Secondary Haversian system, lamellar bone, and lacunae are visible in the majority of the thin  section. Carbon deposition in midcortical. Large | | No grinding scratches. | |
| LC10 | | 3,7 | | Macroscopically, it appears greyish-white throughout the section. Microscopic appearance is dark brown. Secondary Haversian system, lamellar bone, and lacunae are visible in the majority of the thin section. Carbon deposition in periosteal and midcortical, especially in lacunae. Large and microcracks are present, along with the osteon splitting phenomenon. | | Presence of grinding scratches. | |
| LC11 | | 4,1 | | Macroscopically, it appears brownish throughout the section. Microscopic appearance is tan to brown. Secondary Haversian system, lamellar bone, and lacunae are visible in the majority of the thin section. Carbon deposition in periosteal and  midcortical. Large cracks and the osteon splitting phenomenon are present. | | Presence of grinding scratches. | |
| LC12 | | 4,5 | | Macroscopically, it appears greyish-white throughout the section. Microscopic appearance is tan to brown. Secondary Haversian system, lamellar bone, and lacunae are visible in the majority of the thin section. Light carbon deposition in midcortical.  Large and microcracks are present, along with the osteon splitting phenomenon. | | Presence of grinding scratches. | |
| LC13 | | 3,1 | | Macroscopically, it appears brownish throughout the section. Microscopic appearance is tan to brown. Secondary Haversian system, lamellar bone, and lacunae are visible in the majority of the thin section. Lacunae are partially obliterated. No  carbon deposition. Large and microcracks are present, along with the osteon splitting phenomenon. | | Presence of grinding scratches. | |
| LC14 | | 4,7 | | Macroscopically, it presents the so-called sandwich effect. Microscopic appearance is tan in the periosteal to brown in the midcortical. Secondary Haversian system and lacunae are visible in the majority of the thin sections. Secondary Haversian system, lamellar bone, and lacunae are visible in the majority of the thin section. Carbon deposition in  midcortical. Large and microcracks are present, along with the osteon splitting phenomenon. | | No grinding scratches. | |
| LC15 | | 3,7 | | Macroscopically, it appears greyish-white throughout the section. Microscopic appearance is tan to brown. Secondary Haversian system, lamellar bone, and lacunae are visible in the majority of the thin section. Carbon deposition in periosteal and midcortical. Large and microcracks are present, along with the osteon splitting phenomenon. | | No grinding scratches. | |
| LC16 | | 3,3 | | Macroscopically, it appears greyish-white throughout the section. Microscopic appearance is tan to brown. Secondary Haversian system, lamellar bone, and lacunae are visible in the majority of the thin section. Light carbon deposition in midcortical.  Large and microcracks are present, along with the osteon splitting phenomenon. | | No grinding scratches. | |
| LC17 | | 3,1 | | Macroscopically, it appears greyish-white throughout the section. Microscopic appearance is dark brown. Secondary Haversian system, lamellar bone, and lacunae are visible in the majority of the thin section. Carbon deposition in periosteal and midcortical, especially in lacunae. Large cracks and  the osteon splitting phenomenon are present. | | No grinding scratches. | |

| LC18 | 3,6 | Macroscopically, it appears greyish-white throughout the section. The microscopic appearance is brown.  Secondary Haversian system, lamellar bone, and lacunae are visible in the majority of the thin section. Carbon deposition in midcortical,  especially in lacunae. Large and microcracks are present, along with the osteon splitting phenomenon. | No grinding scratches. |
| --- | --- | --- | --- |

# **Supplementary References**

1. Alteration of Bone from Three Archaeological Sites. Journal of Archaeological Science. 1995;22(2):201-9. doi: https://doi.org/10.1006/jasc.1995.0022
