# Supplementary figures and images for: Macroscopic and histological analyses of cremated remains from the Imperial Roman necropolis of La Cona (1st cent. BCE-1st cent. CE, Teramo, Italy)
Source: PLoS One. 2026 Apr 22;21(4):e0345498. doi: 10.1371/journal.pone.0345498 (PMC13102198; doi:10.1371/journal.pone.0345498)

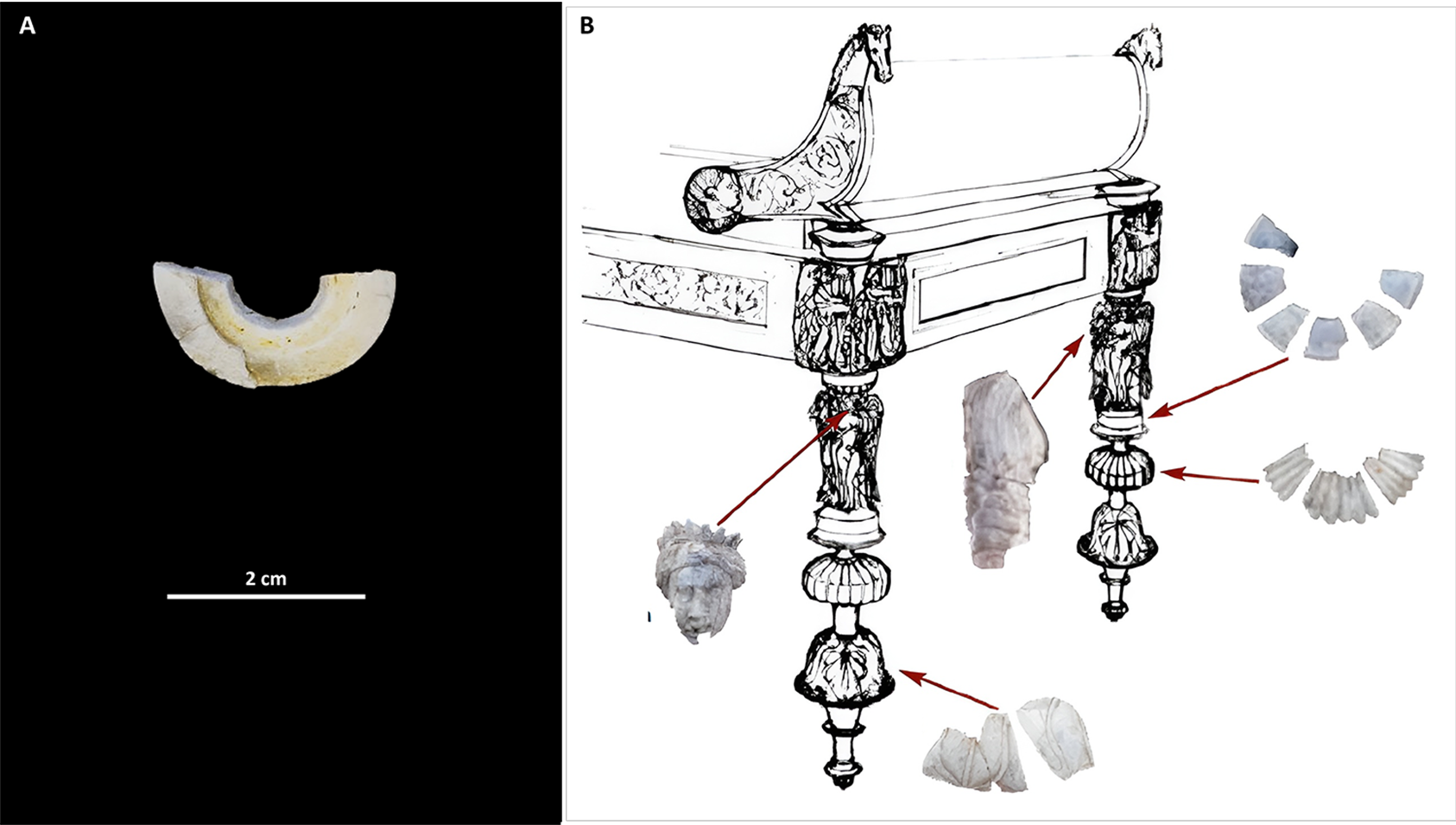

Supplement: S1 Fig — (TIF) [file pone.0345498.s006.tif]

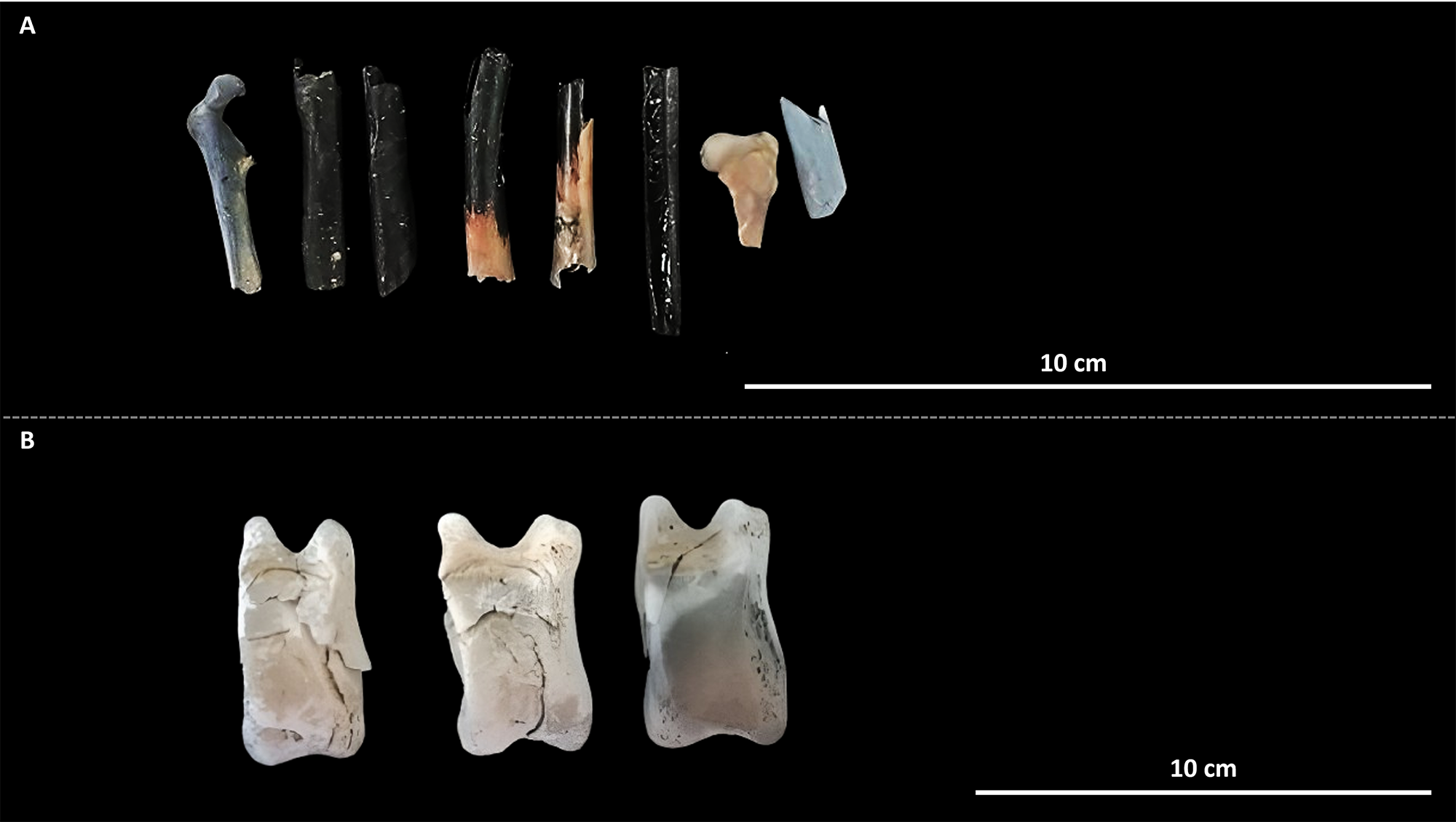

Supplement: S2 Fig — (TIF) [file pone.0345498.s007.tif]
